# Parliament and the Professions

**Published:** 1919-08-30

**Authors:** 


### PARLIAMENT AND THE PROFESSIONS.

### National Health Insurance.

Major Astor informed Mr. G. Locker-Lampson that the question of what changes, if any, in the existing medical benefits under the Insurance Act will be proposed for 1920 is not yet decided. Various improvements in the conditions of service and the possible establishment of some of the additional matters that were suggested in the Estimates of 1914 have been under consideration by the Ministry and in conference with the practitioners, and will be further discussed with the Consultative Councils, with the Insurance Committee, and with Approved Societies.

### Smallpox.

It was stated on behalf of the Ministry of Health that during the month of July and the first week of August sixty cases of smallpox have come to the knowledge of the Ministry. Of these, one group of cases, numbering twenty-five up to August 7, is associated with the return home of a sailor, and three groups, numbering eight cases in all up to August 7, to soldiers returning home from abroad. There have been three ship-borne cases intercepted by Port Sanitary Authorities, from which there has been no spread of infection.

